# Epidrugs: alternative chemotherapy targeting *Theileria annulata* schizont stage parasites

**DOI:** 10.1128/spectrum.03258-23

**Published:** 2024-02-29

**Authors:** Sonam Kamble, Sakshi Singh, Akash Suresh, Siva Singothu, Debabrata Dandesena, Vasundhra Bhandari, Paresh Sharma

**Affiliations:** 1 National Institute of Animal Biotechnology, Hyderabad, India; 2 Graduate Studies, Regional Centre for Biotechnology (RCB), Faridabad, India; 3 National Institute of Pharmaceutical Education and Research, Hyderabad, India; University of Manitoba, Winnipeg, Manitoba, Canada

**Keywords:** epigenetic inhibitors, *Theileria*, drug repurposing, apoptosis, apicomplexan

## Abstract

The growing emergence of resistance to current anti-theilerial agents necessitates the exploration of alternative approaches to drug discovery. This study evaluated the antiparasitic efficacy of 148 compounds derived from an epigenetic inhibitor library against the schizont stage of a *Theileria annulata*-infected cell line. Initial screening at a concentration of 10 µM identified 27 compounds exhibiting promising anti-theilerial activity. Further investigation, including determination of the 50% inhibitory concentration (IC_50_) and host cell cytotoxicity assay, highlighted seven highly effective compounds (SAHA, BVT-948, Trichostatin A, Methylstat, Plumbagin, Ryuvidine, and TCE-5003) against *T. annulata*-infected cells. Analysis of the active compounds revealed their inhibitory action against various human targets, such as HDAC (SAHA and Trichostatin A), SET domain (Ryuvidine), PRMT (BVT-948 and TCE-5003), histone demethylase (Methylstat), and ROS/apoptosis inducer (Plumbagin). We identified gene orthologs of these targets in *Theileria* and conducted molecular docking studies, demonstrating effective binding of the compounds with their respective targets in the parasite, supported by *in vitro* data. Additionally, we performed *in silico* ADME/T predictions, which indicated potential mutagenic and hepatotoxic effects of Plumbagin, Methylstat, and TCE-5003, rendering them unsuitable for drug development. Conversely, SAHA, Trichostatin A, and BVT-948 showed promising characteristics and may represent potential candidates for future development as chemotherapeutic agents against tropical theileriosis. These findings provide valuable insights into the search for novel anti-theilerial drugs and offer a basis for further research in this area.

**IMPORTANCE:**

*Theileria annulata* is a protozoan parasite responsible for tropical theileriosis, a devastating disease affecting cattle. Traditional chemotherapy has limitations, and the study explores the potential of epidrugs as an alternative treatment approach. Epidrugs are compounds that modify gene expression without altering the underlying DNA sequence, offering a novel way to combat parasitic infections. This research is pivotal as it addresses the urgent need for innovative therapies against *T. annulata*, contributing to the development of more effective and targeted treatments for infected livestock. Successful implementation of epidrugs could not only enhance the well-being of cattle but also have broader implications for the control of parasitic diseases, showcasing the paper’s significance in advancing veterinary science and improving livestock health globally.

## INTRODUCTION

*Theileria annulata*, a parasite transmitted by ticks, poses a significant threat to animal health in tropical and subtropical countries, leading to substantial economic losses in the dairy sector (1). The development of resistance to buparvaquone (BPQ), the only available treatment for tropical theileriosis, hinders efforts to eradicate the disease. To combat theileriosis effectively, it is essential to discover new inhibitors or drugs with distinct mechanisms of action that do not share resistance with current therapies. Given the restricted treatment options and the escalating resistance observed in parasites and vectors, the pursuit of novel therapies emerges as a paramount priority (2). One intriguing aspect of apicomplexan parasites is their potential dependence on epigenetic processes to regulate gene expression and cellular development, as they appear to lack the extensive families of identifiable transcription factors found in other eukaryotic species (3). Recent research into the epigenetic gene regulation of apicomplexan parasites like *Plasmodium falciparum* has shown their reliance on epigenetic mechanisms to control various stages of their life cycle (4–8). Similarly, important insights into the epigenetic control of gene expression during the *Theileria* life cycle have been discovered (9, 10).

Currently, drugs targeting epigenetic regulators are under investigation in clinical trials and have been approved by the Food and Drug Administration for cancer therapy (11). Recently, four anti-cancer HDACi [Vorinostat (SAHA), Romidepsin, Belinostat, and Panobinostat] demonstrated anti-theilerial activity against the schizont stage of *T. annulata* parasites (12). To increase the likelihood of identifying effective antiparasitic drugs, the *in vitro* anti-theilerial efficacy of these epigenetic target inhibitors was screened in this study. Additionally, computational approaches were used to identify their target protein in *T. annulata* and evaluate their ADME/T properties (13).

**Fig 1:**
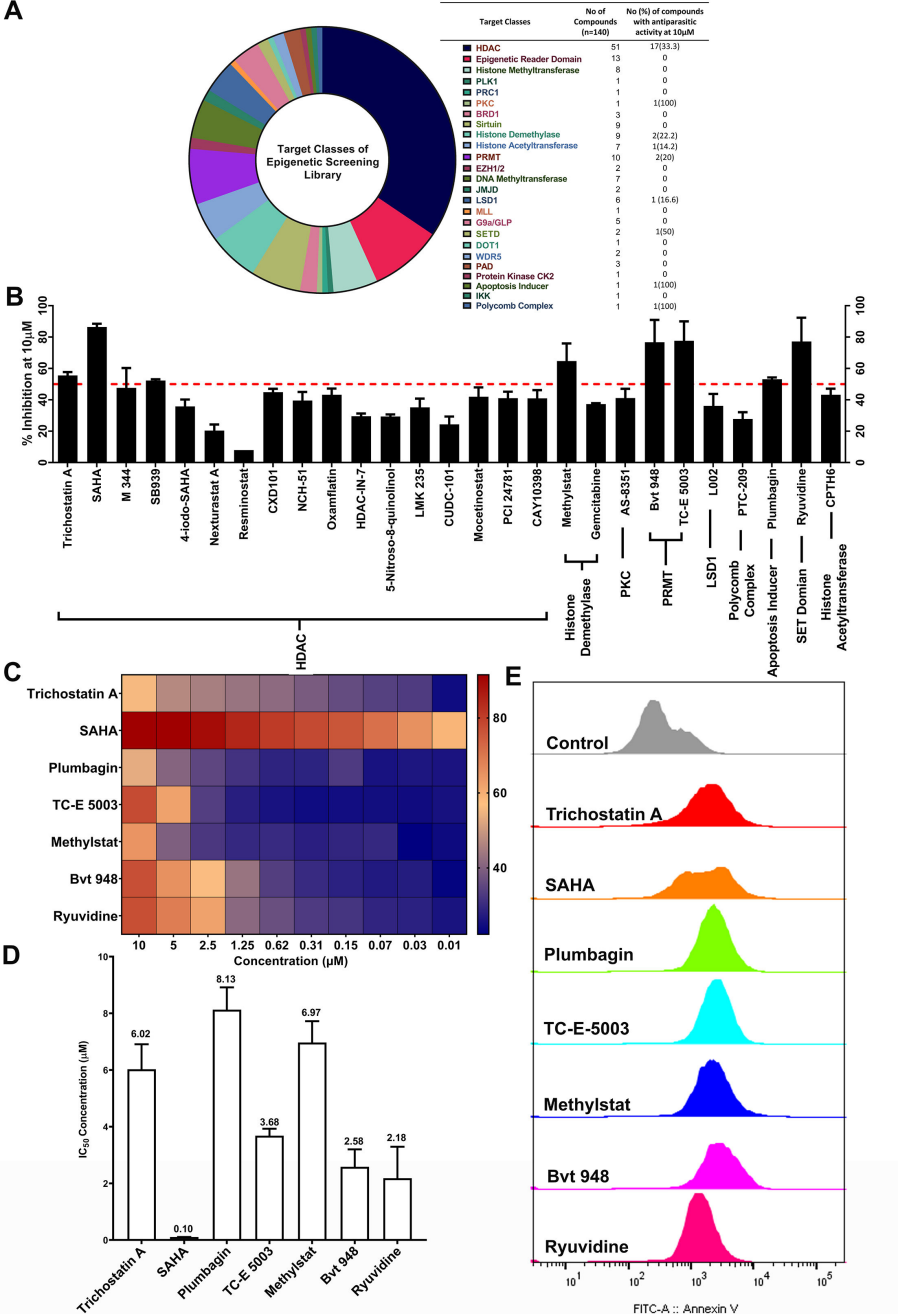
Screening of epigenetic inhibitors for potential anti-theilerial activity in *T. annulata*-infected cells. (**A**) List of compounds and their functional classes used in this study. (**B**) Inhibition (%) of the hits (*n* = 27) with anti-theilerial activity when screened in *T. annulata*-infected cells, where the cut-off (--) was set at 50% inhibition. (**C**) Anti-theilerial activity of best hits that passed the cut-off range, at varying concentrations (μM). (**D**) IC_50_ (μM) of best hits against *T. annulata*-infected cells. (**E**) Flow cytometry-based detection of annexin V-positive *T. annulata*-infected cells upon drug treatment for detection of early apoptosis.

In this study, we conducted a screening of 148 compounds from the Cayman epigenetics library against the asexual schizont stage of *T. annulata*-infected bovine cells to assess their potential anti-theilerial activity (Fig. 1A). The initial screening involved testing the compounds at a concentration of 10 µM, with BPQ serving as the internal control. After 48 hours of pharmacological treatment, 27 compounds demonstrated growth inhibition in *T. annulata*-infected bovine cells (Fig. 1B). Subsequently, we performed drug susceptibility assays on the selected 27 compounds to determine their 50% inhibitory concentration (IC_50_) values against *T. annulata*-infected cells. To do this, 2-fold serial dilutions ranging from 0.019 µM to 10 µM were prepared, and the percentage of cell death was assessed after 48 hours using resazurin-based assays. Based on their IC_50_ values, seven compounds were identified to have potent activity against *T. annulata*-infected cells. Among these, SAHA showed the most potent activity, followed by Ryuvidine, BVT-948, TCE-5003, Trichostatin A, Methylstat, and Plumbagin (Fig. 1C and D). Notably, SAHA's IC_50_ values were comparable to those previously reported by our group for *T. annulata*-infected bovine cells (12) and are further elaborated upon as a positive control. Furthermore, Plumbagin, as recently reported by Titus et al. displayed anti-theilerial effects at a significantly reduced concentration (0.009 µM) compared to our observations (8.13 µM) (14).

To investigate the mechanism of action of these compounds, we evaluated their ability to induce cell death using a propidium iodide (PI) and annexin V-based FACS apoptosis assay. After 48 hours of treatment, all the compounds showed annexin V-positive and PI-negative cells, indicating typical early apoptotic markers (Fig. 1E). No evidence of necrosis was observed in the treated samples, as indicated by the absence of PI-positive cells, suggesting that apoptosis is the primary cause of death after drug treatment. Importantly, none of the seven compounds exhibited cytotoxicity on the bovine macrophage cell line or peripheral blood mononuclear cells isolated from bovine blood when treated for 48 hours at a concentration of 10 µM.

We used Z-scores-based Tanimoto coefficients to understand the structural similarities among the seven lead molecules (15). The Z-scores for all the compounds ranged between 0.7 and 0.9, indicating significant structural variation among the chemicals studied and distinguishing them from BPQ. Notably, Methylstat and Plumbagin showed the highest Z-score of 0.91, while Plumbagin and BVT-948 exhibited the lowest value of 0.62. These compounds that displayed activity against asexual parasites target different epigenetic factors in humans, including HDAC (SAHA and Trichostatin A), a SET domain (Ryuvidine), PRMT (BVT-948 and TCE-5003), histone demethylase (Methylstat), and ROS and apoptosis inducer (Plumbagin).

Next, we aimed to investigate the binding of the seven compounds with their respective targets in *T. annulata* by checking the orthologs of human targets in the parasite. To accomplish this, we identified human orthologs of HDAC (TA12690; 59% homology), Sir2 (TA20415; 37% homology), PRMT (TA20055; 33% homology), and Jmj-ZnF (TA19925; 33% homology) within *T. annulata*. However, since the three-dimensional structures of these proteins were unavailable, homology modeling was conducted to predict their structures. The modeled proteins were validated using Ramachandran plots, and the results showed that over 95% of the amino acids were present in the allowed region, indicating reliable protein models (16). Further, molecular docking was performed to assess the interactions between the compounds and the *T. annulata*-specific target proteins (Table 1). The docking involved the following ligand-protein interactions: (a) SAHA and Trichostatin A with HDAC(TA12690), (b) Plumbagin with Sir2 protein (TA20415), (c) BVT-948 and TCE-5003 with PRMT (TA20055), and (d) Methylstat with Jmj-ZnF (TA19925) in an extra precision mode. The docking results for all ligands concerning their respective proteins are documented in Table 1. SAHA and Trichostatin A showed favorable docking scores against TA12690 and formed metal coordination with a Zinc ion present within the protein, which contributed to their inhibitory action. Additionally, both SAHA and Trichostatin A formed the same interaction bond with the GLY85 amino acid residue of TA12690. Owing to Trichostatin A's specificity toward type 1 histone deacetylases (HDACs) in contrast to SAHA, a pan-HDAC inhibitor, it is plausible that this disparity may elucidate the heightened antiparasitic efficacy exhibited by SAHA relative to Trichostatin A. For Plumbagin and BVT-948, good docking scores were observed with TA20415 and TA20055 proteins, respectively. Plumbagin established hydrogen bonds with ARG68, GLN211, and ASN212, and a pi-pi stacking interaction with the amino acid residue HIS231, suggesting stability and inhibitory action against the Sir2 protein (TA20415) of *T. annulata*. On the other hand, BVT-948 formed hydrogen bonds only with GLU445 and LYS446 of the PRMT protein (TA20055). Furthermore, Methylstat exhibited a docking score of −4.22 kcal/mol with Jmj-ZnF, forming hydrogen bonds with SER280 and HIS370, and engaging in pi-pi stacking with TYR273 and PHE281. These interactions indicated potential inhibitory activity against the TA19925 protein of *T. annulata*. All the identified hits underwent assessment for their ADME/T properties, and it was found that Methylstat violated Lipinski's rule of 5. Moreover, Methylstat exhibited lower oral absorption percentage and Caco cell permeability compared to the other inhibitors. On the other hand, Trichostatin A and Plumbagin demonstrated a high percentage of oral absorption, exceeding 80%. However, when the toxicity profile of these compounds was predicted using the pkCMS Web server, it indicated that Plumbagin might cause mutations, and TCE-5003 and Methylstat exhibited a hepatotoxic nature (Table 1). Further, the study will be expanded in the future by performing *in vitro* experiments based on our data, where we intend to show their parasite-specific killing mechanisms. In conclusion, our study suggests that Trichostatin A and BVT-948 hold promise as potential drugs to combat *T. annulata* infection. Earlier investigations have highlighted the anti-plasmodial activities for both Trichostatin A (5) and BVT-948 (17). Our results confirm and expand on previous research that focused on individual epigenetic enzyme classes. We demonstrated that inhibitors targeting different epigenetic pathways can effectively inhibit the survival of *Theileria*-infected cells, making them potential candidates for developing new anti-theilerial drugs or for use in combination with existing therapies.

**TABLE 1:** Details of molecular docking and ADME/T profile of the six compounds

Molecular docking					ADME properties using QikProp					Toxicity profile by pkCSM	
Protein name	Epigenetic inhibitors	Docking score (kcal/mol)	Interaction type	Residues	Molecular weight (<500 Da)	Rule of 5	QPlogS (predicted aqueous solubility; S in mol/L: −6.5–0.5)	QPPCaco (Caco-2 cell permeability in nm/s:<25 – poor, >500 – great)	Percent human oral absorption (80% is high)	AMES toxicity	Hepato-toxicity
TA12690	SAHA	−8.544	Hydrogen bond	GLY85	264.324	0	−1.781	129.6	69.2	No	No
			Metal coordination	Zinc							
	TSA	−8.239	Hydrogen bond	GLY85, HIE114	302.372	0	−3.859	230.9	80.3	No	No
			Metal coordination	Zinc							
TA20415	Plumbagin	−5.455	Hydrogen bond	ARG68, GLN211, ASN212	188.182	0	−0.493	1434.7	84.2	Yes	No
			PI-PI stacking	HIS231							
TA20055	BVT-948	−6.059	Hydrogen bond	GLU445, LYS446	241.246	0	−2.658	311.9	75.4	No	No
	TCE-5003	−4.338	Hydrogen bond	LYS446, MET473, GLH507	401.264	0	−5.264	232.7	82.3	No	Yes
			PI-PI stacking	PHE366							
TA19925	Methylstat	−4.223	Hydrogen bond	SER280, HIS370	505.569	0	−6.275	20.1	58.9	No	Yes
			PI-PI stacking	TYR273, PHE281							
